# Albumin kinetics, intravascular fluid volume, and respiratory function in pigs ventilated at different levels of mechanical power following crystalloid vs. albumin infusion

**DOI:** 10.1186/s40635-026-00853-0

**Published:** 2026-02-05

**Authors:** Simone Gattarello, Gaetano Gazzé, Emanuele Rollo, Beatrice Donati, Martina Caronna, Ilaria Grava, Carlo Chiumiento, Zhe Li, Walter Gallese, Domenico Nocera, Stefano Giovanazzi, Aurelio Sonzogni, Chiara Sonzogni, Alessandro Gatta, Francesca Collino, Luigi Camporota, Michael Quintel, Onnen Moerer, Federica Romitti, Luciano Gattinoni, Mattia Busana

**Affiliations:** 1 Department of Anesthesiology, University Medical Centre Göttingen, Robert Koch Straße 40, 37075 Göttingen, Germany; 2 Department of Anesthesiology, Critical Care and Pain Medicine, “La Sapienza” University of Rome, Piazzale Aldo Moro, 5, 00185 Rome, Italy; 3 Department of Precision and Regenerative Medicine and Jonica Area, University of Bari “Aldo Moro”, Piazza Umberto I, 70121 Bari, Italy; 4 Department of Anesthesia and Intensive Care, Policlinico di Bari, Piazza Giulio Cesare 11, 70124 Bari, Italy; 5 Department of Health Sciences, University of Milan, Via Festa del Perdono, 7, 20122 Milan, Italy; 6 Department of Anesthesia, Intensive Care and Emergency, “Città della Salute e della Scienza” Hospital, Corso Bramante, 88, 10126 Turin, Italy; 7 Department of Medicine, Surgery and Dentistry, Scuola Medica Salernitana, University of Salerno, Baronissi Via S. Allende, 43, 84081 Salerno, Italy; 8 Department of Critical Care Medicine, Renji Hospital, School of Medicine, Shanghai Jiao Tong University, Pujian Road 160, Shanghai, 200127 China; 9 Department of Medical and Surgical Sciences, Alma Mater Studiorum, University of Bologna, Via Massarenti 9, 40138 Bologna, Italy; 10 Department of Medical and Surgical Specialties, Radiological Sciences and Public Health, University of Brescia, Piazzale Spedali Civili 1, 25121 Brescia, Italy; 11 Department of Pathology, ASST Bergamo Est, Seriate, Italy; 12 Department of Pediatric Surgery, Spedali Civili di Brescia, Brescia, Italy; 13 Department of Anesthesia and Intensive Care, Ceccarini Hospital, AUSL della Romagna, Riccione, Italy; 14 Centre for Human and Applied Physiological Sciences, School of Basic and Medical Biosciences, King’s College London, London, UK; 15 Guy’s and St Thomas’ NHS Foundation Trust, London, United Kingdom of Great Britain and Northern Ireland

**Keywords:** Fluid balance, Mechanical power, Mechanical ventilation, Albumin, Colloid, Crystalloid, Lung damage

## Abstract

**Background:**

The rationale of albumin use lies in its potential to increase oncotic pressure and optimize tissue perfusion. Randomized trials have not demonstrated a survival benefit, and the effects of albumin on volemia remain unclear. This study investigates, in healthy pigs, the effects of a 48-h albumin infusion on intravascular fluid volume, albumin kinetics, and its impact on respiratory function.

**Methods:**

Thirty-nine healthy female pigs ventilated for 48 h were grouped according to mechanical power (high ~ 18 J/min vs. low ~ 6 J/min) and type of fluid (5% albumin solution vs. crystalloid), generating four experimental groups: MP_LOW_-Crystalloid; MP_LOW_-Albumin; MP_HIGH_-Crystalloid; and MP_HIGH_-Albumin.

**Results:**

Intravascular fluid volume was similar across groups (MP_LOW_-Crystalloid: 1.92 (± 0.38)L; MP_HIGH_-Crystalloid: 1.72 (± 0.40)L; MP_LOW_-Albumin: 1.86 (± 0.37)L; MP_HIGH_-Albumin: 2.10 (± 0.58)L; *p* 0.389). For the same mechanical power, the fraction of albumin lost from the plasma was higher in the albumin compared to the crystalloid groups (MP_LOW_-Albumin: 62 (± 13)% vs. MP_LOW_-Crystalloid: − 16 (± 66)%; and MP_HIGH_-Albumin: 58 (± 24)% vs. MP_HIGH_-Crystalloid: 29 (± 14)%; *p *< 0.001). Albumin groups showed greater ascites (MP_LOW_-Crystalloid: 261 (± 380)mL; MP_HIGH_-Crystalloid: 144 (± 148)mL; MP_LOW_-Albumin: 710 (± 664)mL; MP_HIGH_-Albumin: 685 (± 651)mL; *p* 0.034), and worse end-expiratory lung gas volume and elastance, despite comparable histological damage.

**Conclusions:**

In our cohort, prolonged albumin infusion did not lead to a difference in intravascular fluid volume, but it resulted in the loss of ~ 60% of the infused albumin and ascites development. Ascites was associated with lower end-expiratory lung gas volume and higher elastance, despite similar histological lung damage across the groups.

**Supplementary Information:**

The online version contains supplementary material available at 10.1186/s40635-026-00853-0.

## Background

A key rationale for albumin use lies in its ability to increase plasma oncotic pressure, thereby promoting intravascular volume expansion [1] and reducing peripheral tissue edema [2]. Several physiological studies have investigated the pharmacokinetics of albumin and its impact on fluid distribution, observing an increase in intravascular volume following its administration [3–5]. However, the duration of these studies was limited to 6–8 h; therefore, experimental data on the kinetics and physiological effects of albumin during prolonged infusion are lacking. Conversely, randomized trials with a longer duration of intervention showed no significant differences in mortality or length of hospital stay between albumin and crystalloids [6–8]. The discrepancy between physiological studies and clinical trials highlights the current limitations in our understanding of albumin use: whether bolus or infusion is preferable, the duration of its effectiveness, and the specific clinical conditions in which it may be beneficial.

In a recent experimental animal study, we investigated the respiratory implications of high vs. low fluid balance (FB) combined with high vs. low mechanical power (MP) [9]. Surprisingly, despite all groups showing comparable histological lung damage, those with a high FB had a significantly greater amount of ascites, which resulted in reduced end-expiratory lung gas volume (EEL_G_V) and worsened respiratory system elastance. To achieve the target experimental FB, balanced crystalloid was administered to 50% of the pigs in each of the four experimental groups, while the remaining 50% received a 5% albumin solution. Consequently, the observed effects were independent of the type of fluid used and depended solely on the use of high or low FB, as the proportion of colloid and crystalloid was evenly balanced across all experimental groups.

In this secondary analysis, we grouped the animals based on the type of infused fluid rather than FB, thereby creating four groups of animals with similar FB but exposed exclusively to either albumin or crystalloid. The study hypothesis is that, at equal FB, the use of albumin increases intravascular volume through reduced capillary leakage and potentially minimizing damage to peripheral tissues. The primary objective was to assess intravascular fluid volume (IFV) and albumin kinetics in animals ventilated with low and high MP and receiving either 5% albumin solution or balanced crystalloid, with similar FB, during 48 h. The secondary objective was to assess the impact on respiratory function: lung mechanics and histological damage.

## Materials and methods

Thirty-nine healthy female pigs underwent 48 h of mechanical ventilation. According to the initial study design, each animal was randomized to a specific FB (high vs. low), fluid type (crystalloid vs. 5% albumin solution), and MP (high vs. low) (Figure S1). In the present secondary analysis, animals were grouped according to the applied MP and fluid type, resulting in four experimental groups: (1) low mechanical power-crystalloid (MP_LOW_-Crystalloid), *n* = 10; (2) low mechanical power-albumin (MP_LOW_-Albumin), *n* = 9; 3) high-mechanical power-crystalloid (MP_HIGH_- Crystalloid), *n* = 10; and 4) high-mechanical power-albumin (MP_HIGH_-Albumin), *n *= 10. The detailed description of the experimental trial is reported in the online supplement. The study was approved by the local ethics committee (LAVES 33.9-42,502-04-22-00022), and was performed in accordance with the ARRIVE guidelines [10].

### Experimental mechanical power

The values of MP used to define low- and high-MP groups were identified based on the available literature [11–13]: ~ 6 J/min vs. ~ 18 J/min for the low- and the high-MP groups, to provide “safe” or “harmful” ventilation. We maintained a fixed PEEP value (7 cm H₂O) and respiratory rate (15 bpm), and to achieve the targeted MP, tidal volume was adjusted (~ 8 mL/kg in low MP vs. ~ 16 mL/kg in high MP). Once the experimental mechanical ventilation was initiated, the setting of the ventilator was maintained unmodified throughout the experiment.

The calculation of MP was performed according to its simplified equation [14]:

1 $$\mathrm{MP}=0.098\times \mathrm{RR}\times {V}_{t}\times \left({P}_{\mathrm{peak}}- \frac{{P}_{\mathrm{driv}}}{2}\right)$$

where 0.098 is the unit conversion factor; RR is the respiratory rate, *V*_t_ is the tidal volume; *P*_peak_ is the peak pressure; and ^*P*^_driv_ is the driving pressure.

### Experimental fluid type and fluid balance

The study animals were allocated to either a balanced crystalloid solution (Sterofundin^®^, Braun, Melsungen, Germany), or a 5% albumin solution diluted in Sterofundin^®^. The 5% albumin-crystalloid solution was prepared by removing 250 mL from 1L bag of Sterofundin^®^ and replacing it with 250 mL of 20% albumin (Human Albumin Grifols^®^ 200 g/L).

Although in the original trial animals were grouped into low FB (increase of 0–5% in body weight at 48 h compared to baseline) or high FB (increase of 10–15%), regardless of the type of fluid, in this secondary analysis animals were grouped according to the type of fluid. Consequently, FB is anticipated to be comparable among groups.

### Assessment of intravascular fluid volume

The IFV was computed at baseline (IFV_BL_) and at each timepoint of the study (IFV_X_).

The IFV_BL_ was computed as the 5% of the initial body weight:

2 $${\mathrm{IFV}}_{\mathrm{BL}}={\mathrm{TBW}}_{\mathrm{BL}}\text{ x }0.05$$

where TBW_BL_ is the baseline total body weight.

The IFV_X_ was calculated using the mass balance equation based on hemoglobin concentration. The first step consisted in the computation of baseline amount of hemoglobin, according to the following equations:

3 $${[\mathrm{Hb}]}_{\mathrm{BL}}= \frac{{\mathrm{qHb}}_{\mathrm{BL}}}{{\mathrm{IFV}}_{\mathrm{BL}}}$$

4 $${\mathrm{qHb}}_{\mathrm{BL}} ={[\mathrm{Hb}]}_{\mathrm{BL}}\text{ x }{\mathrm{IFV}}_{\mathrm{BL}}$$

where [Hb]_BL_ is the baseline concentration of hemoglobin; qHb_BL_ is the baseline amount of hemoglobin within the IFV_BL_; and IFV_BL_ is the baseline intravascular fluid. Rearranging Eq. 3 to isolate qHB_BL_ as the unknown variable (Eq. 4), we obtained the baseline hemoglobin amount (qHb_BL_).

Second, we corrected for the hemoglobin removed at each timepoint of the study, based on the volume of blood lost due to blood sampling, to obtain the amount of hemoglobin within the IFV (qHb_X_). Hence, we calculated the IFV at each timepoint of the study (IFV_X_), using the following equation:

5 $${\mathrm{IFV}}_{\mathrm{X}}= \frac{{\mathrm{qHb}}_{\mathrm{X}}}{{[\mathrm{Hb}]}_{\mathrm{X}}}$$

where qHb_X_ is the amount of hemoglobin within the IFV at each timepoint of the study; and [Hb]_X_ is the hemoglobin concentration at each timepoint.

### Quantification of the expected amount of albumin

The expected albumin (qAlb_EXP_), i.e., the amount of protein within the IFV assuming no albumin loss, was calculated as the sum of the baseline albumin (qAlb_BL_) plus the amount of infused albumin at each study timepoint.

The baseline amount of albumin (qAlb_BL_) was computed as follows:

6 $${\mathrm{qAlb}}_{\mathrm{BL}} ={[\mathrm{Alb}]}_{\mathrm{BL}}\text{ x }{\mathrm{IFV}}_{\mathrm{BL}}$$

where [Alb]_BL_ is the albumin concentration at baseline; and IFV_BL_ is the baseline IFV.

To quantify the expected albumin (qAlb_EXP_) at each timepoint, we summed the qAlb_BL_ with the albumin infused at each timepoint, which was derived by quantifying the infused milliliters of 5% albumin solution.

### Quantification of the actual amount of albumin and wasted albumin

Once the value of IFV was computed for each study timepoint, the actual amount of albumin (qAlb_X_) was derived by applying the following equation:

7 $${\mathrm{qAlb}}_{\mathrm{X}} ={[\mathrm{Alb}]}_{\mathrm{X}}\text{ x }{\mathrm{IFV}}_{\mathrm{X}}$$

where [Alb]_X_ and IFV_X_ are the concentrations of albumin and amount of IFV at a given study timepoint, respectively.

The amount of wasted albumin was determined as follows:

8 $$\mathrm{Alb}.\text{wasted }={\mathrm{qAlb}}_{\mathrm{exp}}- {\mathrm{qAlb}}_{\mathrm{act}}$$

where qAlb_exp_ and qAlb_act_ are the amount of expected and actual albumin at a given study timepoint, respectively.

The fraction of wasted albumin was calculated applying the following equation:

9 $$\mathrm{Alb}.\mathrm{wasted}.\text{fraction }= \frac{\mathrm{Alb}.\mathrm{wasted}}{{\mathrm{qAlb}}_{\mathrm{exp}}}\text{ x }100$$

### Study process

Once the animals were prepared for the experimental setting, baseline measurement was performed, in pre-experimental conditions. Subsequently, the setting of mechanical ventilation, the infusion rate and the type of fluid to be infused were modified, according to the group allocation and the study protocol. A complete set of measurements was collected at baseline, at 0.5 h, and subsequently every 6 h. At 48 h, the animal was euthanized and the autopsy performed. Macroscopic and microscopic lung analyses were performed to quantify the magnitude of lung damage.

### Outcome variables

All data related to pulmonary and cardiac functions, together with fluid distribution, were collected: time course of EEL_G_V, partitioned respiratory mechanics, hemodynamic and fluid-related variables. We recorded the amount of infused and eliminated fluids and albumin. At each measurement, we collected blood samples for lab analysis and gas exchange assessment. Post-mortem, we measured lung weight and ascites and collected tissue samples from the lung, liver, kidney, bowel, and muscle for wet-to-dry ratio assessment and histological analysis of lung tissue.

### Statistical analysis

Data were expressed as mean ± SD or percentage. Comparison between groups and subgroups was performed using one-way ANOVA or Fisher's exact test. Time course of the collected variables was evaluated via a two-way ANOVA for repeated measures with group as between effect, time as a within effect, and the single animal as random effect. A *p* value < 0.05 was considered statistically significant. All analyses were performed with R 4.0.

## Results

At baseline, physiological variables were comparable across groups, except for wedge pressure and pulmonary vascular resistances (Table S1). The volume of fluids administered, the FB, and the amount of infused albumin were similar between the crystalloid and albumin groups, for the same MP, as per study protocol (Table 1 and Figure S2).

**Table 1:** End-experimental values for the assessed variables according to the four experimental groups

	MP_LOW_-crystalloid	MP_LOW_-albumin	MP_HIGH_-crystalloid	MP_HIGH_-albumin	*p* value
Fluid distribution					
Weight [kg]	33.1 (± 4.1)	33.2 (± 3.7)	35.5 (± 7.4)	35.1 (± 3.9)	0.621
Weight variation [kg]	2.42 (± 1.42)	2.05 (± 1.73)	2.02 (± 1.50)	2.38 (± 2.15)	0.931
Infused fluids [L]	2.79 (± 1.71)	2.69 (± 2.45)	3.16 (± 2.39)	3.13 (± 2.40)	0.956
Infused albumin [g]	0	135 (± 122)	0	156 (± 120)	< 0.001
Wasted albumin [g]	− 2.8 (± 12.8)	116.1 (± 89.5)	4.8 (± 3.5)	119.2 (± 96.1)	< 0.001
Wasted albumin [%]	− 16 (± 66)	62 (± 13)	29 (± 14)	58 (± 24)	0.002
Urine production [L]	1.26 (± 0.48)	1.81 (± 0.85)	1.85 (± 0.87)	1.67 (± 0.86)	0.337
Fluid balance [L]	2.53 (± 1.44)	2.21 (± 1.64)	2.35 (± 1.70)	2.55 (± 1.70)	0.962
Ascites [mL]	261 (± 380)	710 (± 664)	144 (± 148)	685 (± 651)	0.034
IFV [L]	1.92 (± 0.38)	1.86 (± 0.37)	1.72 (± 0.40)	2.10 (± 0.58)	0.389
Albumin in IFV [g]	18.6 (± 13.9)	52.1 (± 31.6)	10.4 (± 2.9)	52.4 (± 31.7)	0.002
Hemoglobin in IFV [g]	146.2 (± 35.5)	144.2 (± 19.0)	143.4 (± 36.3)	163.3 (± 42.9)	0.545
Hemodynamics					
Heart rate [bpm]	67 (± 36)	72 (± 25)	71 (± 16)	92 (± 36)	0.280
Mean systemic pressure [mmHg]	61 (± 10)	67 (± 12)	62 (± 12)	78 (± 15)	0.019
Central venous pressure [mmHg]	9 (± 6)	7 (± 6)	9 (± 5)	13 (± 5)	0.128
Mean pulmonary pressure [mmHg]	15 (± 4)	19 (± 6)	21 (± 6)	25 (± 6)	0.014
Pulmonary wedge pressure [mmHg]	9 (± 5)	10 (± 4)	12 (± 4)	18 (± 10)	0.019
Cardiac output [L/min]	2.97 (± 1.41)	4.10 (± 1.74)	2.80 (± 0.81)	2.97 (± 1.57)	0.198
SVR [dyn·sec·cm⁻^5^]	1601 (± 513)	1171 (± 278)	1626 (± 667)	1715 (± 1112)	0.357
PVR [dyn·sec·cm⁻^5^]	214 (± 95)	175(± 76)	264(± 195)	219(± 210)	0.685
SvO_2_ [%]	61.6 (± 15.2)	67.6 (± 8.5)	53.2 (± 5.9)	55.6 (± 14.1)	0.057
Ventilator setting					
Tidal volume [mL]	261 (± 35)	269 (± 22)	541 (± 67)	559 (± 70)	< 0.001
Respiratory rate [bpm]	15 (± 0)	15 (± 0)	15 (± 0)	15 (± 0)	1.000
PEEP [cmH_2_O]	6.7 (± 0.5)	6.7 (± 0.5)	7.4 (± 1.1)	7.4 (± 1.1)	0.088
FiO_2_ [%]	40 (± 0)	40 (± 0)	40 (± 0)	40 (± 0)	1.00
Lung mechanics					
Mechanical power [J/min]	6.98 (± 2.90)	7.75 (± 1.86)	22.54 (± 5.38)	26.48 (± 7.59)	< 0.001
EEL_G_V [mL]	739 (± 158)	755 (± 213)	1047 (± 296)	867 (± 241)	0.037
Elastance [cmH_2_O/L]	40.2 (± 9.8)	45.6 (± 9.3)	34.5 (± 10.5)	41.1 (± 13.7)	0.193
Gas exchange and laboratory tests					
pH	7.48 (± 0.06)	7.47 (± 0.06)	7.58 (± 0.02)	7.52 (± 0.09)	0.003
PaCO_2_ [mmHg]	42.0 (± 9.0)	49.0 (± 8.1)	19.6 (± 3.8)	24.6 (± 9.4)	< 0.001
PaO_2_ [mmHg]	170.5 (± 33.0)	154.2 (± 27.3)	177.2 (± 43.5)	161.9 (± 34.1)	0.528
Hemoglobin [g/dL]	7.6 (± 0.9)	7.6 (± 0.9)	8.1 (± 1.3)	8.2 (± 2.2)	0.749
Plasma albumin [g/dL]	0.92 (± 0.50)	2.56 (± 1.30)	0.64 (± 0.19)	2.30 (± 1.06)	< 0.001
Urinary albumin [mg/L]	4.4 (± 3.2)	29.9 (± 27.6)	5.6 (± 4.8)	16.8 (± 12.4)	0.005

Values are expressed as mean (SD); *p* value: one-way ANOVA; *IFV* Intravascular fluid volume, *SVR* Systemic vascular resistances, *PVR* Pulmonary vascular resistances, *SvO*_*2*_ Mixed venous oxygen saturation, *PEEP* Positive end-expiratory pressure, *FiO*_*2*_ Fraction of inspired oxygen, *EEL*_*G*_*V* End-expiratory lung gas volume, *PaCO2* Arterial partial pressure of carbon dioxide and *PaO2* arterial partial pressure of oxygen

### Intravascular fluid volume

**Fig. 1:**
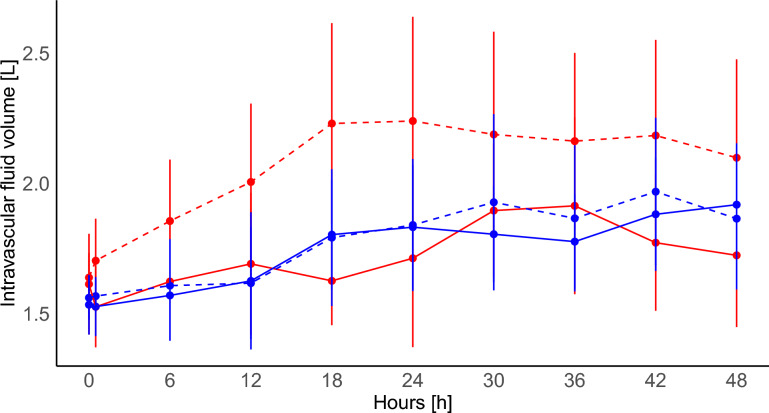
Time course of intravascular fluid volume across the four experimental groups: MP_LOW_-Crystalloid: solid blue line; MP_LOW_-Albumin: dashed blue line; MP_HIGH_-Crystalloid: solid red line; MP_HIGH_-Albumin: dashed red line. Two-way ANOVA: differences between groups *p* 0.095; differences over time *p *< 0.001; interaction group-time: *p* 0.817

No significant differences in IFV were observed over time across groups (Fig. 1), or at 48 h: MP_LOW_-Crystalloid: 1.92 (± 0.38)L; MP_LOW_-Albumin: 1.86 (± 0.37)L; MP_HIGH_-Crystalloid: 1.72 (± 0.40)L; MP_HIGH_-Albumin: 2.10 (± 0.58)L; *p* 0.389 (Table 1). The MP_HIGH_-Albumin group demonstrated a non-significant trend of higher IFV values, with the largest gap relative to the other groups occurring between hours 12 and 24.

### Dynamics of albumin: plasma kinetics and clearance

**Fig. 2:**
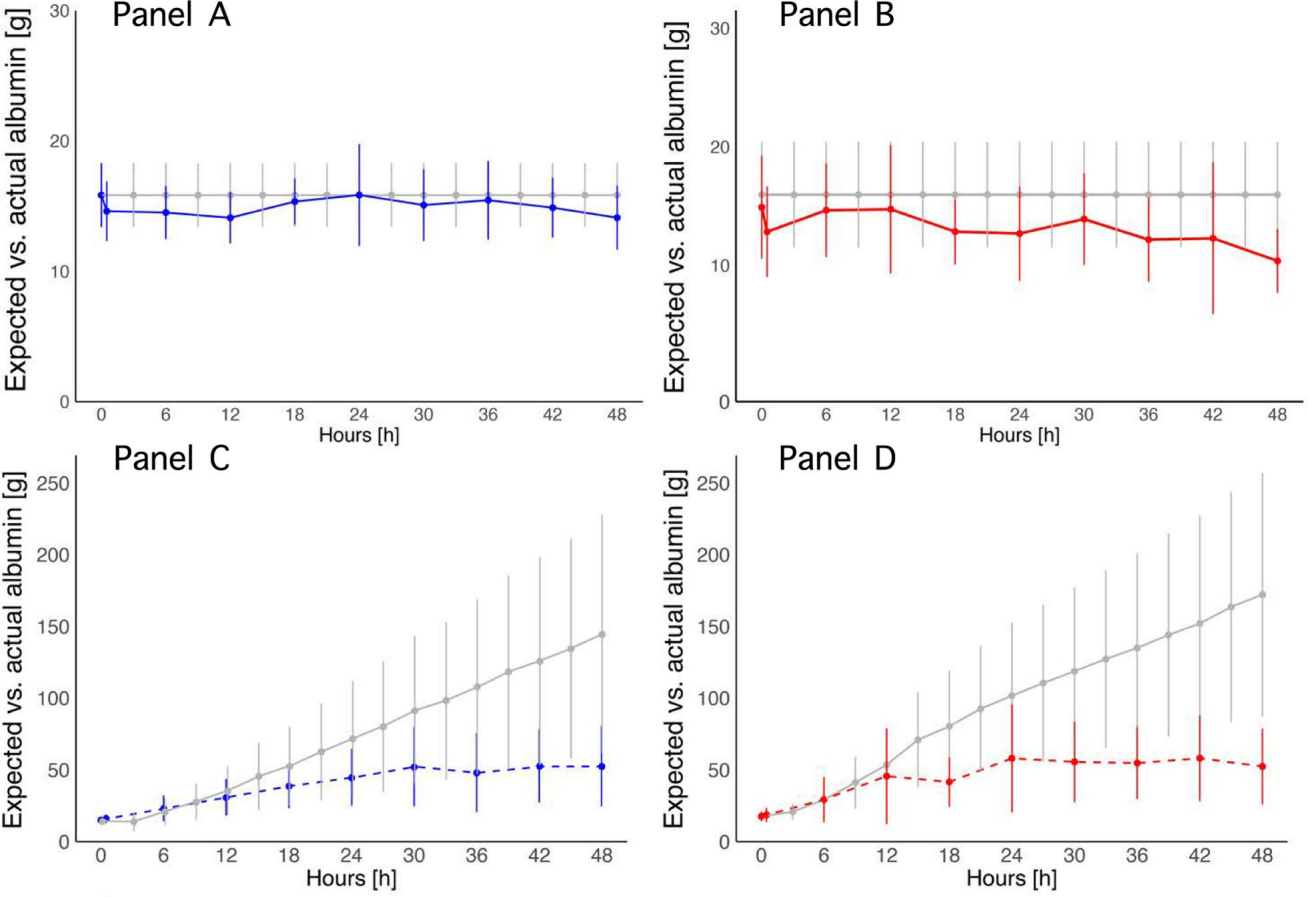
Time course of expected (gray line) and actual albumin (blue or red line) in the four experimental groups. Panel** A** MP_LOW_-Crystalloid, solid blue line, *p* interaction 0.370; Panel** B** MP_HIGH_-Crystalloid, solid red line, *p* < 0.001; Panel **C** MP_LOW_-Albumin, dashed blue line, *p* < 0.001; Panel** D**, MP_HIGH_-Albumin: dashed red line, *p* < 0.001

**Fig. 3:**
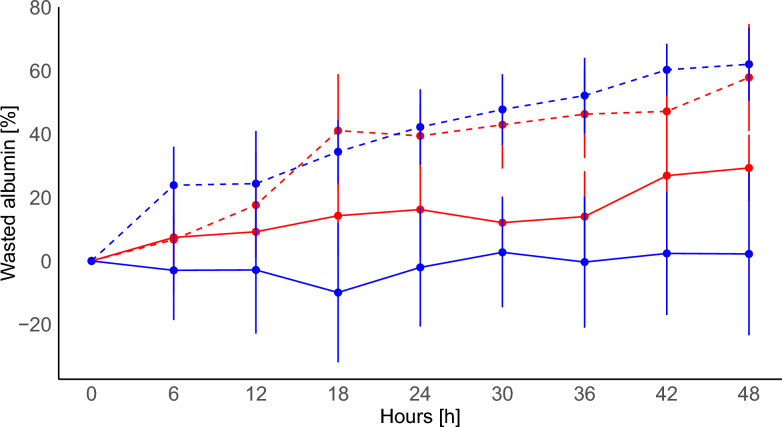
Time course of wasted albumin fraction in the four experimental groups: MP_LOW_-Crystalloid: solid blue line; MP_LOW_-Albumin: dashed blue line; MP_HIGH_-Crystalloid: solid red line; MP_HIGH_-Albumin: dashed red line. Two-way ANOVA: differences between groups *p* 0.695; differences over time *p* 0.557; interaction group-time: *p* < 0.001

Figure 2 shows the expected amount of albumin that would be present in the circulatory system in the absence of any removal from circulation (qAlb_exp_), compared to the actual amount of albumin (qAlb_act_). While groups assigned to crystalloid showed a minimal difference between expected and actual albumin levels (Panel A and B), the albumin groups showed a substantial and significant gap (Panel C and D). As a result, the groups assigned to albumin had a significantly higher amount of wasted albumin (Fig. 3); at 48 h: MP_LOW_-Albumin: 62 (±13)%, and MP_HIGH_-Albumin: 58 (±24)%. Conversely, in the crystalloid groups, wasted albumin showed smaller variations: MP_LOW_-Crystalloid: − 16 (± 66)%, and MP_HIGH_-Crystalloid: 29 (±14)% (difference between the four groups *p *< 0.001; Table 1).

**Fig. 4:**
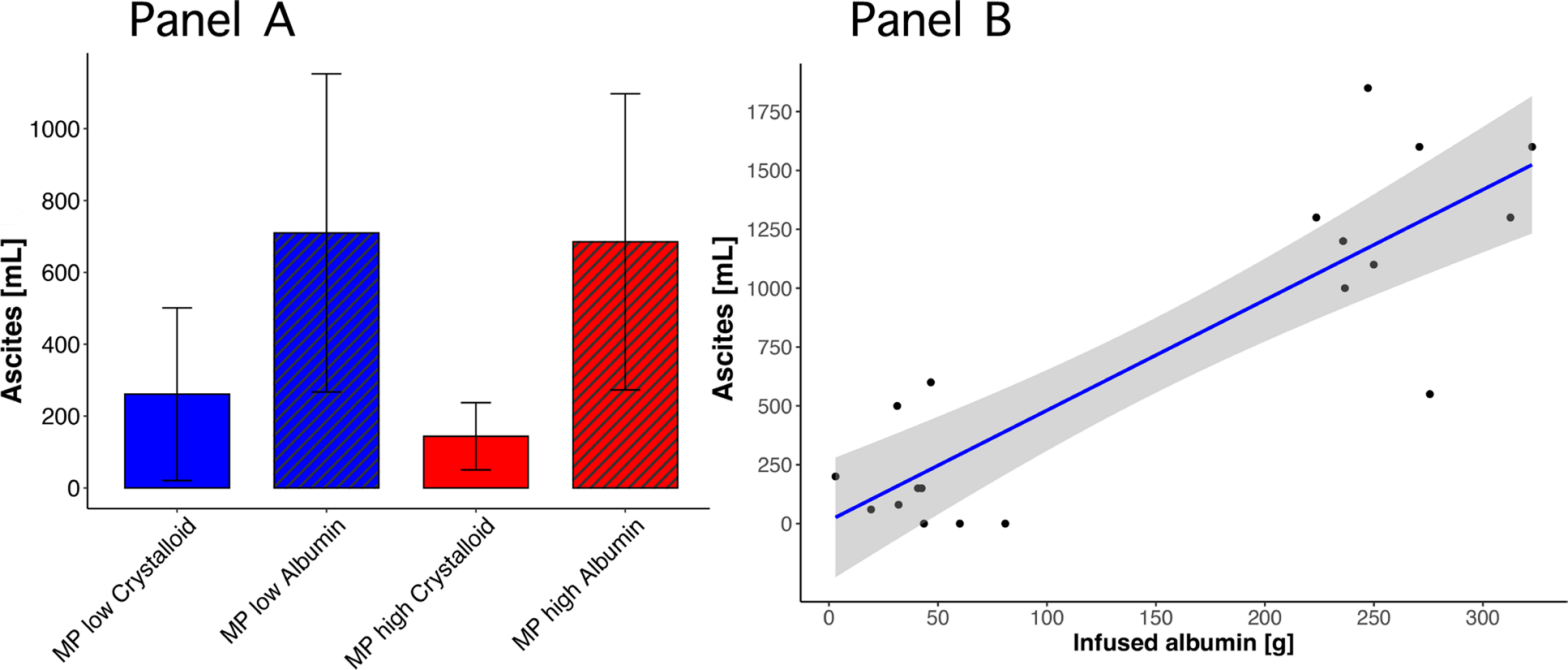
Panel** A** End-experimental amount of ascites in the four experimental groups: MP_LOW_-Crystalloid: solid blue bar; MP_LOW_-Albumin: striped blue bar; MP_HIGH_-Crystalloid: solid red bar; MP_HIGH_-Albumin: striped red bar. One-way ANOVA: *p* 0.034. Panel **B** association between the end-experimental amount of infused albumin and ascites, in animals assigned to albumin. *p* < 0.001; *R*^2^ = 0.739; β = 4.298

The groups receiving albumin had a higher volume of ascites: MP_LOW_-Crystalloid: 261 (± 380)mL; MP_LOW_-Albumin: 710 (± 664)mL; MP_HIGH_-Crystalloid: 144 (± 148)mL; MP_HIGH_-Albumin: 685 (± 651)mL; *p* 0.034 (Fig. 4, Panel A). In a linear regression model, the amount of infused albumin showed a significant linear relationship with increased ascites (*p* < 0.001; *R*^2^ = 0.750; β = 4.686; Fig. 4, Panel B).

### Respiratory function: lung mechanics and histological damage

**Fig. 5:**
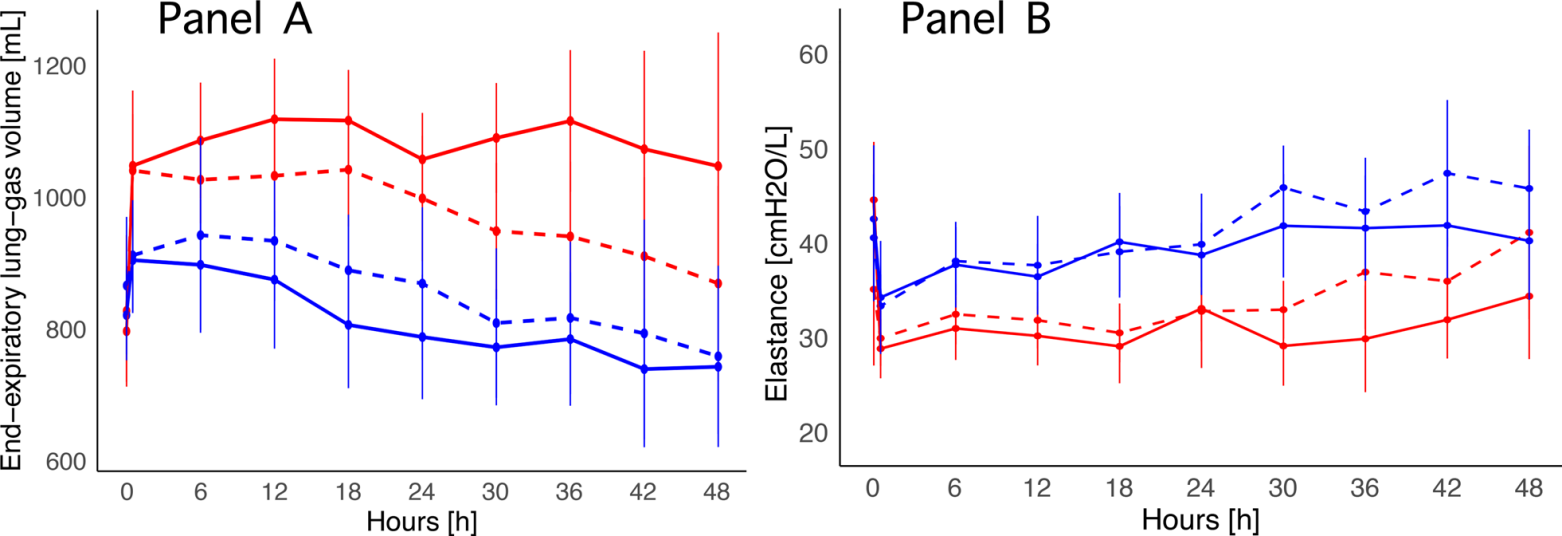
Time course of end-expiratory lung gas volume (Panel A), and respiratory system elastance (Panel B) in the four experimental groups: MP_LOW_-Crystalloid: solid blue line; MP_LOW_-Albumin: dashed blue line; MP_HIGH_-Crystalloid: solid red line; MP_HIGH_-Albumin: dashed red line. Panel** A** (two-way ANOVA): differences between groups *p* 0.044; differences over time *p *< 0.001; interaction group-time: *p* 0.008. Panel** B** (two-way ANOVA): differences between groups *p* 0.022; differences over time *p* 0.023; interaction group-time: *p* 0.582

Measured EEL_G_V throughout the experiment is shown in Fig. 5, Panel A. In groups assigned to low MP, the relative variation in EEL_G_V was similar: MP_LOW_-Crystalloid: −18.7 (± 20.3)%; MP_LOW_-Albumin: −16.4 (± 23.8)% (Table 1). Conversely, when assessing the groups assigned to high MP, the difference was pronounced: the crystalloid group maintained a stable EEL_G_V, while the group assigned to albumin showed a reduction in EEL_G_V, comparable to that of the low-MP groups: MP_HIGH_-Crystalloid: 6.2 (± 30.6)%; MP_HIGH_-Albumin: −16.4 (± 21.1)% (difference between the four experimental groups: *p* 0.034).

The groups with the highest reduction in EEL_G_V showed the steepest increase in respiratory system elastance, particularly during the last 24 h. Conversely, the high-MP crystalloid group, which had a stable EEL_G_V, maintained a constant pulmonary elastance over time (Fig. 5, Panel B). No significant differences between groups were observed in lung weight, wet-to-dry ratio, or the incidence of histopathological alterations, as reported in Table 2.

**Table 2:** Post-mortem analysis: macroscopic and microscopic indicators of lung damage

	MP_LOW_-crystalloid	MP_LOW_-albumin	MP_HIGH_-crystalloid	MP_HIGH_- albumin	*p* value
Macroscopic pathology					
Lung weight [mg]	482.4 (± 179.4)	518.7 (± 176.4)	593.5 (± 197.0)	588.3 (± 261.2)	0.570
Wet-to-dry ratio: lung	6.06 (± 0.68)	6.20 (± 0.74)	6.47 (± 0.75)	6.71 (± 0.92)	0.275
Wet-to-dry ratio: liver	4.25 (± 0.65)	3.89 (± 0.46)	4.30 (± 0.52)	4.31 (± 0.60)	0.340
Wet-to-dry ratio: kidney	5.97 (± 0.64)	5.75 (± 0.85)	5.99 (± 0.75)	5.67 (± 0.65)	0.705
Wet-to-dry ratio: bowel	5.03 (± 1.23)	5.25 (± 0.64)	5.45 (± 0.57)	5.32 (± 0.57)	0.687
Wet-to-dry ratio: muscle	4.26 (± 0.53)	4.25 (± 0.35)	4.07 (± 0.33)	3.94 (± 0.37)	0.245
Microscopic pathology					
Alveolar ruptures [%]*	100	100	100	100	1.000
Alveolar edema [%] *	90	77.8	80	80	0.947
Inflammation [%]*	100	100	100	100	1.000
Atelectasis [%]*	100	100	90	100	1.000

Values are expressed as mean (SD); *p* value: one-way ANOVA; * values expressed as a percentage of the total observations; *p* value: Fisher's exact test

## Discussion

This study investigated the effects of albumin infusion on intravascular fluid volume, albumin kinetics, and respiratory function compared to crystalloid, for the same fluid balance, in healthy pigs ventilated at two MP levels. The main findings were: (1) intravascular fluid volume was similar between the experimental groups and, in albumin groups, the amount of wasted albumin was as high as ~ 60%; (2) albumin infusion was associated with significantly higher ascites volume; and (3) ascites was associated with reduced EEL_G_V and worse respiratory mechanics, despite similar histological lung damage. Taken together, our results do not support the original study hypothesis.

### Intravascular fluid volume

Intravascular fluid volume was similar across groups, regardless of the type of fluid infused, despite a transient and non-significant trend toward higher IFV in the MP_HIGH_-Albumin group.

Although our findings do not align with previous physiological studies reporting an increase in plasma volume following albumin infusion [3–5], two key considerations should be taken into account: (1) the duration of this study was longer, whereas previous studies had a limited duration of a few hours. The MP_HIGH_-Albumin group had the highest IFV, likely due to the combination of higher oncotic pressure (from albumin infusion) and increased intrathoracic pressures (from MP), especially during the 12–24-h period, while in the subsequent hours, the gap with the other groups decreased. It is possible that a different duration of the previously reported studies would have yielded different results. Similarly, in the ALBIOS trial, individuals assigned to albumin had higher mean arterial pressure and central venous pressure during the first days of the trial, while in the following days, the values became similar between groups [6]. (2) The majority of studies supporting the positive effects of albumin on IFV administered it as a bolus, whereas in this study, we used continuous infusion. In patients undergoing major abdominal surgery, Statkevicius et al. observed that the same amount of albumin administered as a bolus had a greater plasma-expanding effect compared to continuous infusion [15]. Therefore, the use of different infusion rates may in part explain the heterogeneity of results observed in studies evaluating the effects of albumin.

### Albumin kinetics and albumin waste

In the groups assigned to albumin, a significant difference between expected and actual albumin levels was observed, confirming a substantial albumin loss from the intravascular space. In contrast, actual albumin levels in the crystalloid groups remained relatively stable, consistent with minimal loss from catabolism, dilution, or third-space shift [16]. In the low-MP crystalloid group, the negative value of albumin waste is unlikely to reflect a true increase in total plasma albumin content. Rather, this may be explained by a lower intensity of mechanical ventilation leading to reduced activation of volume-regulating pathways (renin–angiotensin–aldosterone system, antidiuretic hormone, etc.) and, in turn, less water reabsorption and less “dilution” compared to the high-MP group crystalloid [17].

As shown in Fig. 1, in the albumin groups, expected and actual albumin overlap during the initial 6–8 h and subsequently diverge, suggesting the existence of a threshold beyond which albumin is no longer effective and albumin waste occurs. Although albumin concentration in ascitic fluid was not measured, it is reasonable to speculate that albumin shifted into this compartment. This hypothesis is supported by the positive association between infused albumin and ascites volume, as well as by the absence of ascites in the crystalloid groups. Two potential mechanisms could explain ascites development: (1) in an initial phase, ascites may have formed through a passive mechanism dependent on increased intravascular hydrostatic pressure and (2) subsequently, an oncotic gradient-driven accumulation of fluids, where increased albumin in the peritoneal space may have raised the intraperitoneal oncotic pressure above plasma oncotic pressure, drawing fluids from the intravascular compartment into the peritoneal space [18].

To investigate the mechanisms underlying albumin loss, we constructed several linear regression models in which different variables were tested against the amount of wasted albumin (Figures S4–S8). In light of these results, albumin loss was most strongly associated with the amount of albumin infused, consistently across both low- and high-mechanical power groups. According to Starling’s principle, the movement of fluid and protein, including albumin, across the capillary wall depends on the balance of hydrostatic and oncotic pressures and capillary permeability [19]. As our animals did not have conditions associated with increased permeability, albumin extravasation is likely driven by alterations in hydrostatic or oncotic pressures. After leaving the capillary, albumin is normally reabsorbed via the lymphatic system and returned to the circulation. However, human [20] and animal [21] studies show that increased central venous pressure impairs lymphatic flow, thereby reducing the reabsorption of interstitial fluid and its protein content. The differing association between central venous pressure and albumin loss in albumin- vs. crystalloid-treated animals ventilated at high-mechanical power supports this proposed mechanism. Moreover, in our model, higher plateau and driving pressures were associated with greater albumin loss, likely reflecting the increase in central venous pressure induced by elevated intrathoracic pressures [22].

### Lung mechanics and lung pathology

Different MP and fluid types resulted in similar macroscopic and microscopic histological lung damage. This finding confirms the observations from our previous study, where we observed that high vs. low MP and FB were associated with comparable lung injury.

Although evidence supports that high MP is associated with greater lung injury, reconciling the observation of lung injury at low MP is more challenging. In our previous paper, we proposed that overdistention and volutrauma were the main contributors in high-MP groups, whereas derecruitment and atelectrauma were the primary mechanisms when low MP was applied. Another possible hypothesis is that 48 h may be insufficient to observe different lung injuries across groups, as lung damage from volutrauma and atelectrauma might develop with different timing and latency. On the other hand, the use of albumin vs. crystalloid did not significantly affect lung injury severity, and the most likely explanation is that albumin waste, probably into the ascitic fluid, neutralized the anti-edema effects of albumin.

Although histological damage was similar across experimental groups, animals assigned to albumin showed reduced EEL_G_V and worse respiratory system elastance. As thoroughly discussed in our previous paper, the primary factor contributing to the reduction in EEL_G_V was the development of ascites, resulting from increased intra-abdominal pressure and displacement of the diaphragm [23].

### Limitations

The present study has several limitations. The fluid management does not reflect the dynamic approach currently used in clinical practice, but was designed to investigate the effects on fluid distribution and lung injury resulting from different combinations of MP and fluid type. Ascites was only quantified post-mortem and not throughout the experiment, and we were unable to measure albumin concentration in ascites or intra-abdominal pressure; therefore, our interpretation remains speculative. The different MP was generated by only modifying the tidal volume. However, this is not a mere comparison between different tidal volumes, as the same tidal volumes could result in different levels of injury under varying experimental conditions, such as varying respiratory rate or PEEP [24]. Although the mass balance equation is the most commonly used method for quantifying plasma volume in studies examining the effects of albumin infusion, its accuracy may decline over time.

## Conclusions

In the present study, the initial hypothesis that prolonged albumin infusion would increase IFV was not confirmed. Indeed, in groups assigned to albumin, nearly 60% of the infused albumin was wasted, i.e., removed from the intravascular compartment, and likely shifted into the peritoneal space. In contrast, animals receiving crystalloids neither showed albumin waste nor developed ascites. The presence of ascites was associated with impaired lung mechanics, despite the absence of significant differences in histological lung injury across groups. Although these results cannot be directly applied to daily clinical practice, they provide relevant insights into albumin kinetics in the context of mechanical ventilation.

## Supplementary Information

Supplementary Material 1.

## Data Availability

The data that support the findings of this study are available from the corresponding author, upon reasonable request.

## Abbreviations

FB: Fluid balance
MP: Mechanical power
EEL_G_V: End-expiratory lung gas volume
IFV: Intravascular fluid volume
