# Supplementary material for: Albumin kinetics, intravascular fluid volume, and respiratory function in pigs ventilated at different levels of mechanical power following crystalloid vs. albumin infusion
Source: Intensive Care Med Exp. 2026 Feb 5;14:12. doi: 10.1186/s40635-026-00853-0 (PMC12872945; doi:10.1186/s40635-026-00853-0)
Supplement: Supplementary file 1 — Supplementary Material 1. [file 40635_2026_853_MOESM1_ESM.docx]

**Albumin kinetics, fluid distribution, and respiratory function in pigs ventilated at different levels of mechanical power following crystalloid vs. albumin infusion**

Simone Gattarello MD, PhD; Gaetano Gazzé MD; Emanuele Rollo MD; Beatrice Donati MD; Martina Caronna MD; Ilaria Grava MD; Carlo Chiumiento MD; Zhe Li MD, PhD; Walter Gallese MD; Domenico Nocera MD, Stefano Giovanazzi MD; Aurelio Sonzogni MD; Chiara Sonzogni; Alessandro Gatta MD; Francesca Collino MD; Luigi Camporota MD, PhD; Michael Quintel MD; Onnen Moerer MD; Federica Romitti MD^^^; Luciano Gattinoni MD^^^; and Mattia Busana MD

**ONLINE DATA SUPPLEMENT**

**Corresponding author:**

Simone Gattarello MD, PhD. Department of Anesthesiology, University Medical Center Gottingen, Robert Koch Straße 40, 37075 Goettingen, Germany. E-mail: gattarello@gmail.com

**Supplemental methods**

Thirty-nine female pigs undergoing 48 hours long mechanical ventilation were grouped according to the level of applied mechanical power (MP) (High versus Low) and the type of fluid infused (5% albumin solution versus balanced crystalloid solution). Therefore, the four experimental groups were the following: 1) Low Mechanical Power-Crystalloid (MP_LOW_-Crystalloids), n=10; 2) Low Mechanical Power-Albumin (MP_LOW_-Albumin), n=9; 3) High Mechanical Power-Crystalloid (MP_HIGH_-Crystalloids), n=10; and 4) High Mechanical Power-Albumin (MP_HIGH_-Albumin), n=10.

The experimental trial was previously approved by the local ethics committee (LAVES 33.9-42502-04-22-00022), and was performed in accordance with the ARRIVE guidelines (S1).

*Experimental mechanical power*. The values of low and high MP were chosen based on the available literature (S2-S4) to achieve a power of ~6J/min or ~18J/min in low and high MP groups respectively. The target MP was obtained by setting a tidal volume, in volume-control ventilation, of ~8mL/Kg (low MP) or ~16mL/Kg (high MP), while maintaining a fixed respiratory rate (15/min) and PEEP (7cmH_2_O). Upon the modification of the tidal volume, animals were ventilated for 10 minutes following which the MP was calculated, and tidal volume was adjusted until the target MP was achieved.

Once MP was achieved, the ventilator settings were kept constant throughout the experiment. However, due to the progressive modifications of the anatomical (end-expiratory lung gas volume - EEL_G_V) and physiological (elastance, strain) lung characteristics of the study individuals, the measured MP changed during the experiment.

*Lung mechanics computation.* The ***EEL_G_V*** was provided by the mechanical ventilator (**Carescape, GE Healthcare, Chicago, IL, USA**), which has the capability to calculate it using the **nitrogen dilution method**, relying on the variation of inert gas concentrations in the lungs.

***Respiratory system elastance*** was calculated as follows:

$$E.rs=\frac{Paw.plat-Paw.exp}{Vt}$$

P_aw.plat_: plateau pressure; P_aw.exp_: expiratory pressure; V_t_: tidal volume.

*Experimental fluid type.* The study individuals were randomized to receive either a balanced crystalloid solution (Sterofundin®, Braun, Melsungen, Germania), or a 5% albumin solution diluted in Sterofundin®. The 5% albumin-crystalloid solution was prepared by removing 250 mL from 1L bag of Sterofundin® and replacing it with 250 mL of 20% albumin (Human Albumin Grifols® 200 g/L). Although in the original trial the animals were also randomized to receive low fluid balance (FB) (increase of 0/5% of weight at 48 hours, compared to the baseline) or high FB (increase of 10/15% of weight at 48 hours, compared to the baseline), for the present analysis we only grouped the animals according to the MP and type of fluid, regardless of the FB.

*Study protocol:* Animal randomization, study design, and timeline are reported in Figure S1, Panel A (randomization process) and B (study design).

During the pre-experimental phase, the animals underwent induction of anesthesia, endotracheal intubation, and placement of venous and arterial catheters. Once the animal was optimized for the experiment and before initiation of the experimental phase, the baseline measurement was performed. Subsequently, the setting of ventilator was set to deliver the experimental MP, and the infusion rate and the fluid type were modified to achieve the FB target for the following timepoint of the study.

**Figure S1**: animal randomization (Panel A) and timeline of the experimental trial (Panel B). The randomization process for the present analysis is represented by the groups in the blue box.

**Panel A**


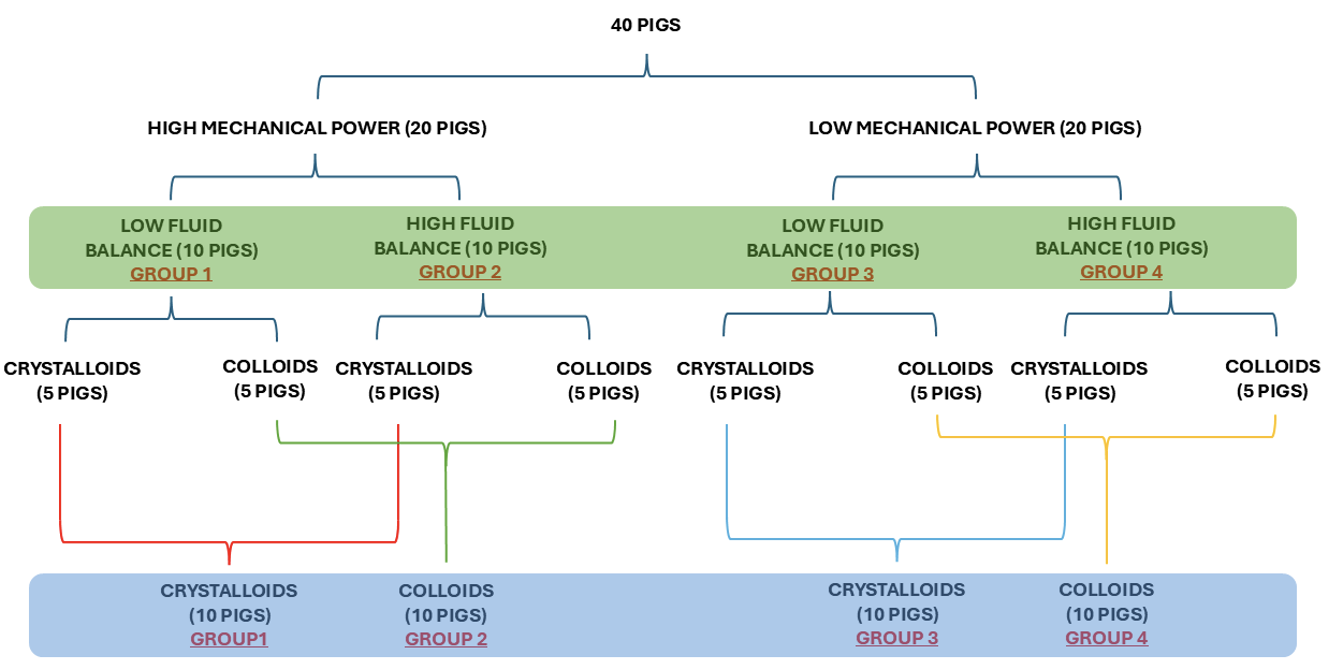


**Panel B**


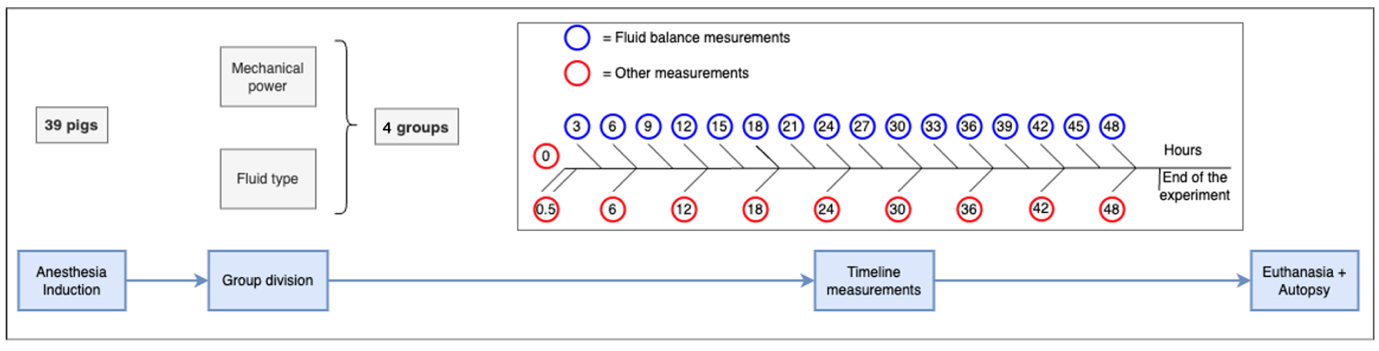


The first measurement was performed at baseline (during standard ventilation, that was equal in all groups); subsequently, it was repeated at 0.5 hours (30 minutes after the application of the experimental protocol) and, afterwards, every 6 hours until the conclusion of the experiment. The FB was computed every 3 hours. At 48 hours, animals were euthanized and the autopsy performed. Lung samples were obtained to perform the wet-to-dry ratio and lung histology.

*Animal preparation:* all animals were premedicated with intramuscular midazolam (5 mg); antibiotic prophylaxis was carried out using intramuscular amoxicillin trihydrate (Duphamox® 16 mg/Kg). After the cannulation of a peripheral vein, induction of anesthesia (azaperone 2 mg/Kg; ketamine 10 mg/Kg; sufentanyl 30 mcg; and propofol 1-2 mg/Kg) and endotracheal intubation were performed. The animals were ventilated in prone position, the natural position of pigs, throughout the study. General anesthesia was managed as follows: propofol 3-5 mg/Kg/h, sufentanyl 1-2 mcg/Kg/h, midazolam 0.8-1.2 mg/Kg/h. The pre-experimental mechanical ventilation setting was the following: tidal volume 8 mL/Kg, respiratory rate to maintain EtCO_2_ 35/40 mmHg, inspiratory to expiratory ratio 1:2, PEEP 4 cmH_2_O, fraction of inspired oxygen 0.40.

Central venous line (5 Fr) and pulmonary artery catheter (5 Fr) were inserted from the left and right jugular veins; the femoral artery was cannulated with 4 Fr arterial line (PiCCO®, monitoring system, Getinge, Sweden). All catheters were placed via ultrasound-guided puncture. A urinary catheter and esophageal pressure nasogastric tube (NutriVentTM, SIDAM S.R.L, Italy) were also positioned. Temperature control was achieved through heating blankets. The animal weight was continuously monitored by a high-precision scale.

*Outcome variables:* all data providing information on the pulmonary function, fluid distribution and circulation were collected: time-course of EEL_G_V, partitioned respiratory mechanics, hemodynamic and fluid-related variables. We recorded the amount of infused and eliminated fluids and albumin. At each measurement, we collected blood samples for lab analysis and gas exchange assessment. The animal weight was assessed at each experimental timepoint by means of a high precision scale. Post-mortem data: absolute lung weight and ascites were quantified; tissue samples of lung, liver, kidney, bowel and muscle were collected for the wet-to-dry analysis, and lung samples were harvested to perform histological analysis.

### *Histology:* the lungs were fixed in 4% formalin for at least one week. Twenty samples of lung tissue were obtained from each animal for histological examination: for each lung, 10 samples were obtained and each one further divided into a dorsal and ventral portion. The samples were fixed, dehydrated in a graded alcohol series, cleared in xylene and embedded in paraffin. Two µm sections were cut with a microtome, mounted on slides, and stained with hematoxylin-eosin for histological analysis. The sections were viewed under a light microscope, and histological scoring was performed by an investigator blinded to the group allocation of the animals. Scoring was attributed with the following methodology: for each one of the variables of interest (alveolar ruptures, alveolar edema, inflammation, atelectasis) the scores of absent (0-25% of the optical field, 2 points), focal (25-50% of the optical field, 4 points), multifocal (50-75% of the optical field, 8 points) and diffuse (75-100% of the optical field, 16 points) were attributed”.

**Supplemental results**

Supplemental tables:

**Table S1.** Baseline values for the assessed variables according to the four experimental groups.

|  | **MP_LOW_-Crystalloids** | **MP_LOW_-Albumin** | **MP_HIGH_-Crystalloids** | **MP_HIGH_- Albumin** | **p value** |
| --- | --- | --- | --- | --- | --- |
| **FLUID DISTRIBUTION** | | | | | |
| **Weight [kg]** | 30.7 (±3.7) | 31.2 (±2.6) | 33.5 (±6.7) | 32.8 (±3.0) | 0.462 |
| **Intravascular fluid volume [L]** | 1.53 (±0.18) | 1.56 (±0.13) | 1.67 (±0.34) | 1.64 (±0.15) | 0.462 |
| **Albumin in IFV [g]** | 15.8 (±3.2) | 15.3 (±3.4) | 14.9 (±5.2) | 17.6 (±4.4) | 0.554 |
| **Hemoglobin in IFV [g]** | 159.9 (±36.9) | 156.4 (±18.8) | 155.2 (±34.4) | 178.6 (±44.4) | 0.423 |
| **HEMODYNAMICS** | | | | | |
| **Heart rate [bpm]** | 74 (±18) | 68 (± 10) | 63 (±21) | 71 (±19) | 0.582 |
| **Mean systemic pressure [mmHg]** | 74 (±9) | 78 (±10) | 75 (±12) | 67 (±6) | 0.101 |
| **Central venous pressure [mmHg]** | 8 (±6) | 6 (±5) | 9 (±3) | 9 (±6) | 0.652 |
| **Mean pulmonary pressure [mmHg]** | 19 (±5) | 18 (±6) | 16 (±3) | 21 (±4) | 0.165 |
| **Pulmonary wedge pressure [mmHg]** | **9 (±4)** | **6 (±3)** | **10 (±3)** | **11 (±3)** | **0.012** |
| **Cardiac output [L/min]** | 2.90 (±0.87) | 3.12 (±0.96) | 2.70 (±1.38) | 3.00 (±0.74) | 0.831 |
| **SVR [dyn·sec·cm⁻⁵]** | 1816 (±695) | 1925 (±702) | 2062 (±631) | 1402 (±540) | 0.139 |
| **PVR [dyn·sec·cm⁻⁵]** | **370 (±101)** | **311 (±85)** | **200 (±75)** | **259 (±102)** | **0.003** |
| **SvO_2_ [%]** | 63.9 (±10.9) | 65.4 (±8.6) | 58.5 (±8.6) | 71.2 (±9.8) | 0.056 |
| **VENTILATOR SETTING** | | | | | |
| **Tidal volume [mL]** | 234 (±26) | 233 (±23) | 227 (±34) | 246 (±28) | 0.528 |
| **Respiratory rate [bpm]** | 21 (±5) | 21 (±6) | 20 (±4) | 21 (±5) | 0.984 |
| **PEEP [cmH_2_O]** | 3.9 (±0.6) | 4.6 (±1.7) | 4.1 (±0.3) | 4.8 (±1.2) | 0.235 |
| **FiO_2_ [%]** | 40 (±0) | 40 (±0) | 40 (±0) | 40 (±0) | 1.00 |
| **LUNG MECHANICS** | | | | | |
| **Mechanical power [J/min]** | 6.42(±2.14) | 6.42(±1.98) | 6.17(±1.88) | 7.29 (±2.81) | 0.698 |
| **EEL_G_V [mL]** | 819 (±112) | 864 (±161) | 794(±125) | 825(±187) | 0.785 |
| **Elastance [cmH_2_O/L]** | 42.49 (±12.26) | 40.55 (±10.02) | 44.46 (±9.65) | 35.23 (±12.69) | 0.309 |
| **GAS EXCHANGE AND LABORATORY TESTS** | | | | | |
| **pH** | 7.47 (±0.04) | 7.44 (±0.05) | 7.47 (±0.06) | 7.46 (±0.08) | 0.761 |
| **PaCO_2_ [mmHg]** | 49.3 (±6.7) | 54.3 (±7.7) | 50.9 (±5.2) | 51.7 (±9.1) | 0.520 |
| **PaO_2_ [mmHg]** | 180.8 (±13.6) | 174.0 (±10.5) | 177.3 (±18.3) | 187.8 (±18.2) | 0.264 |
| **Hemoglobin [g/dL]** | 10.3 (±1.4) | 10.0 (±1.0) | 9.2 (±0.7) | 10.8 (±1.7) | 0.073 |
| **Plasma albumin [g/dL]** | 1.02 (±0.14) | 0.97 (±0.20) | 0.92 (±0.18) | 1.06 (±0.21) | 0.441 |
| **Urinary albumin [mg/L]** | 12.6 (±11.5) | 21.6 (±21.6) | 19.1 (±22.6) | 32.9 (±48.9) | 0.559 |

Values are expressed as mean (SD); p-value: one-way ANOVA; IFV: Intravascular Fluid Volume; SVR: Systemic Vascular Resistances; PVR: Pulmonary Vascular Resistances; SvO_2_: mixed venous oxygen saturation; PEEP: Positive End-Expiratory Pressure; FiO_2_: Fraction of inspired Oxygen; EEL_G_V: End-Expiratory Lung Gas Volume; PaCO2: Arterial Partial Pressure of Carbon Dioxide and PaO2: Arterial Partial Pressure of Oxygen.

Supplemental figures:

**Figure S2.** Time course of infused albumin (Panel A) and mechanical power (Panel B) in the four experimental groups: MP_LOW_-Crystalloids: solid blue line; MP_LOW_-Albumin: dashed blue line; MP_HIGH_-Crystalloids: solid red line; MP_HIGH_-Albumin: dashed red line. Panel A (two-way ANOVA): differences between groups p 0.946; differences over time p 0.148; interaction group-time: p<0.001. Panel B (two-way ANOVA): differences between groups p<0.001; differences over time p<0.001; interaction group-time: p<0.001.

**Figure S3.** Time course of fluid balance in the four experimental groups: MP_LOW_-Crystalloids: solid blue line; MP_LOW_-Albumin: dashed blue line; MP_HIGH_-Crystalloids: solid red line; MP_HIGH_-Albumin: dashed red line. Two-way ANOVA: differences between groups p 0.831; differences over time p<0.001; interaction group-time: p 0.396.

**Analysis of the mechanisms of albumin loss**

The variable showing the strongest statistical association with wasted albumin was the amount of albumin infused during the experiment, both in the low- and high-MP groups (Figure 1A: p <0.001; beta 0.622; R^2^ 0.538; Figure 1B: p <0.001; beta 0.657; R^2^ 0.473).

**Figure S4.** Association between infused and wasted albumin in low- (Figure 1A) and high- (Figure 1B) mechanical power groups. The regression line is shown for the albumin group only, as infused albumin was zero in all crystalloid-treated animals.


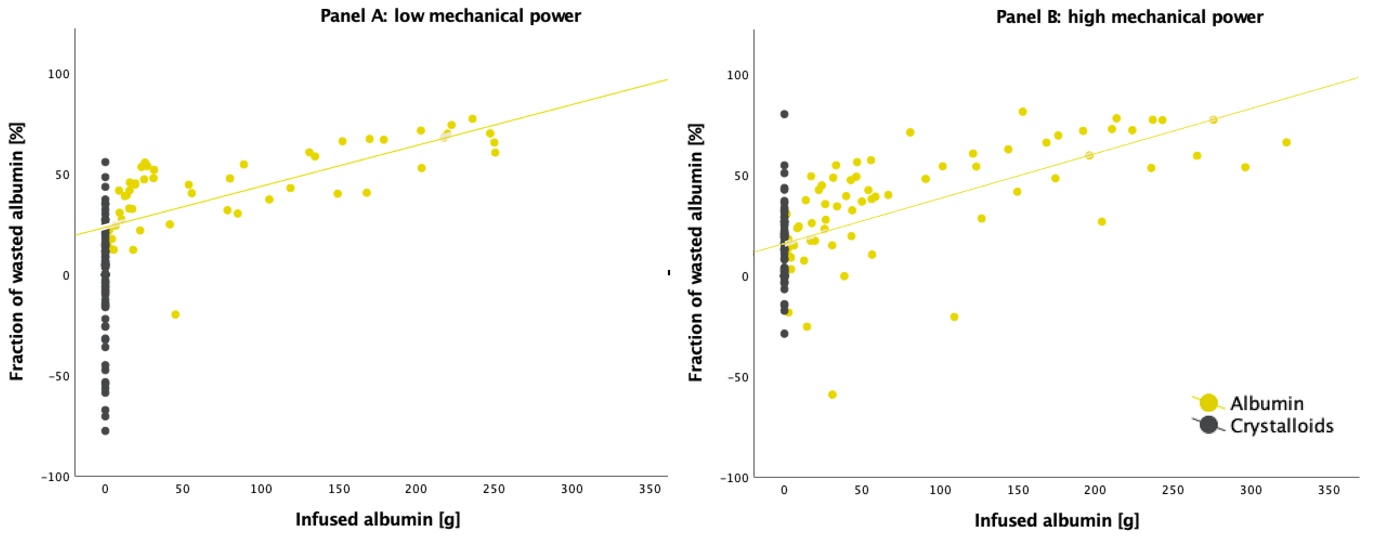


In the low MP groups, changes in CVP were not significantly associated with changes in wasted albumin both in albumin and crystalloids groups (Figure 2A; Albumin: p 0.079; beta -0.234; R2 0.055; Crystalloids: p 0.293; beta -0.119; R2 0.014). In the high mechanical power groups, an increase in CVP was significantly associated with increased wasted albumin in the albumin group, while an opposite trend was observed in the crystalloid group (Figure 2B; Albumin: p 0.003; beta 0.330; R2 0.109; Crystalloids: p <0.001; beta -0.413; R2 0.171).

**Figure S5.** Association between central venous pressure (CVP) and wasted albumin in low- (Figure 2A) and high- (Figure 2B) MP groups.


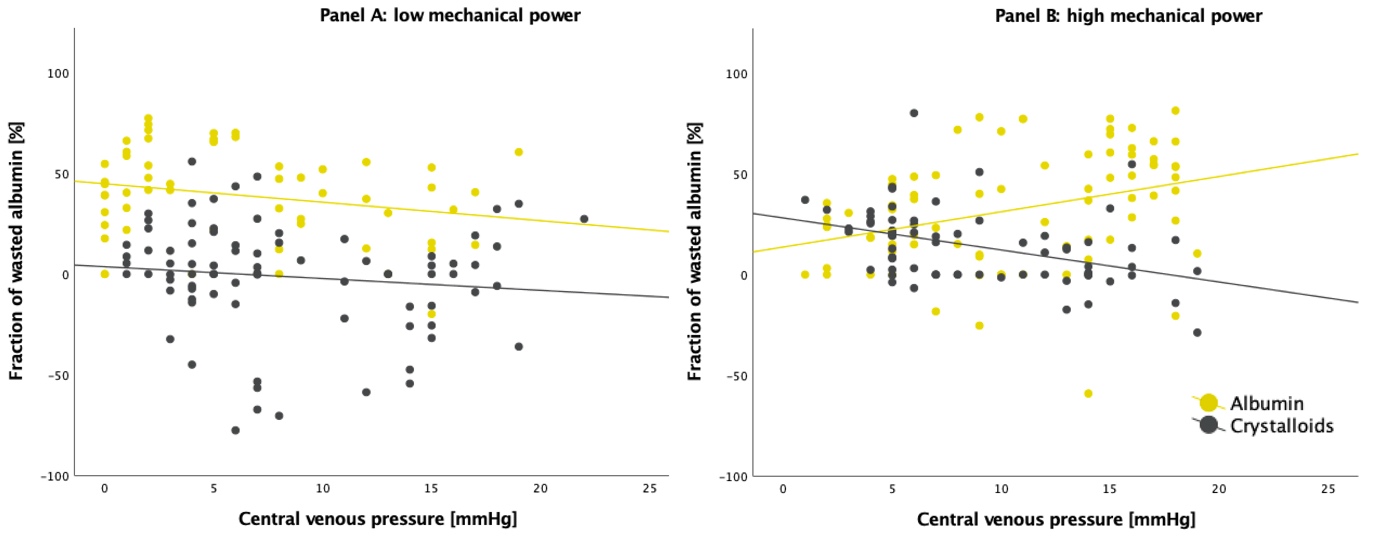


The analysis of cardiac output (CO) did not show statistically significant associations (Figure 3A; Low MP groups: Albumin, p 0.061; beta 0.250; R2 0.063; Crystalloids: p 0.052; beta 0.226; R2 0.051. Figure 3B; High MP groups: Albumin, p 0.362; beta -0.108; R2 0.012; Crystalloids: p 0.318; beta 0.122; R2 0.015).

**Figure S6.** Association between cardiac output (CO) and wasted albumin in low- (Figure 3A) and high- (Figure 3B) mechanical power groups.


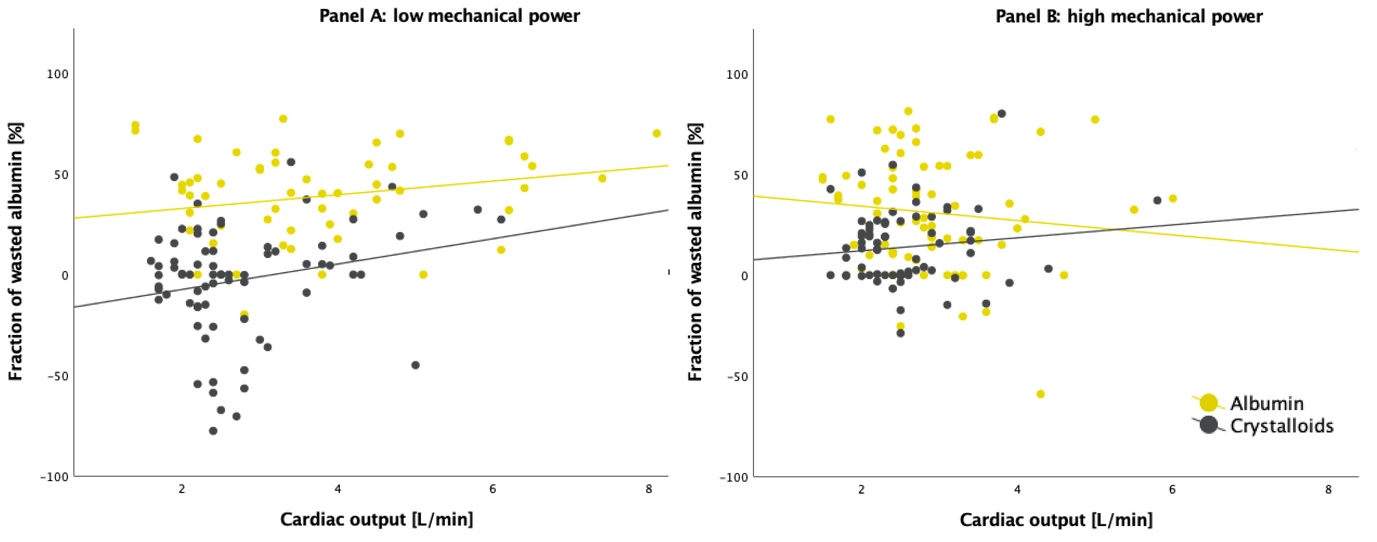


Considering the effects of mechanical ventilation on wasted albumin, in both low- and high-MP groups, increasing plateau pressure (Figures 4) or driving pressure (Figures 5) was associated with a higher fraction of wasted albumin, in animals receiving either albumin or crystalloids (Figure 4A; Low MP groups: Albumin, p <0.001; beta 0.567; R2 0.322; Crystalloids: p <0.001; beta 0.392; R2 0.154. Figures 4B; High MP groups: Albumin, p 0.001; beta 0.377; R2 0.142; Crystalloids: p 0.023; beta 0.271; R2 0.074. Figure 5A; Low MP groups: Albumin, p <0.001; beta 0.524; R2 0.274; Crystalloids: p <0.001; beta 0.499; R2 0.249. Figure 5B; High MP groups: Albumin, p 0.003; beta 0.331; R2 0.109; Crystalloids: p 0.109; beta 0.193; R2 0.037).

**Figure S7.** Association between plateau pressure and wasted albumin in low- (Figure 4A) and high- (Figure 4B) mechanical power groups.

**
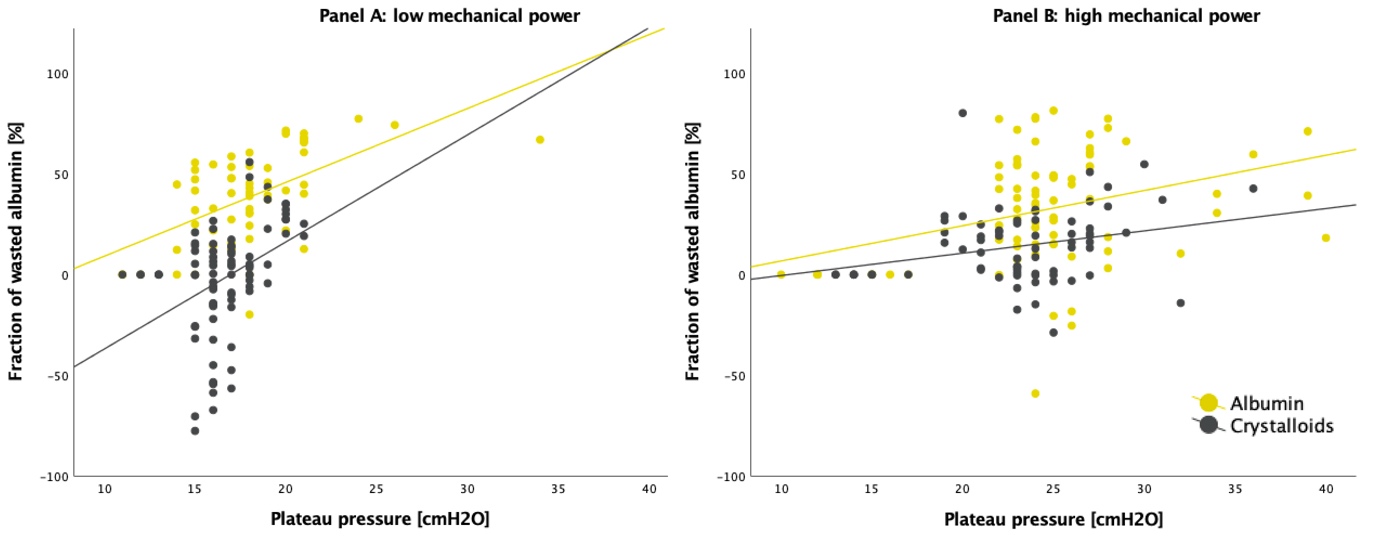
**

**Figure S8.** Association between driving pressure and wasted albumin in low- (Figure 5A) and high- (Figure 5B) mechanical power groups.


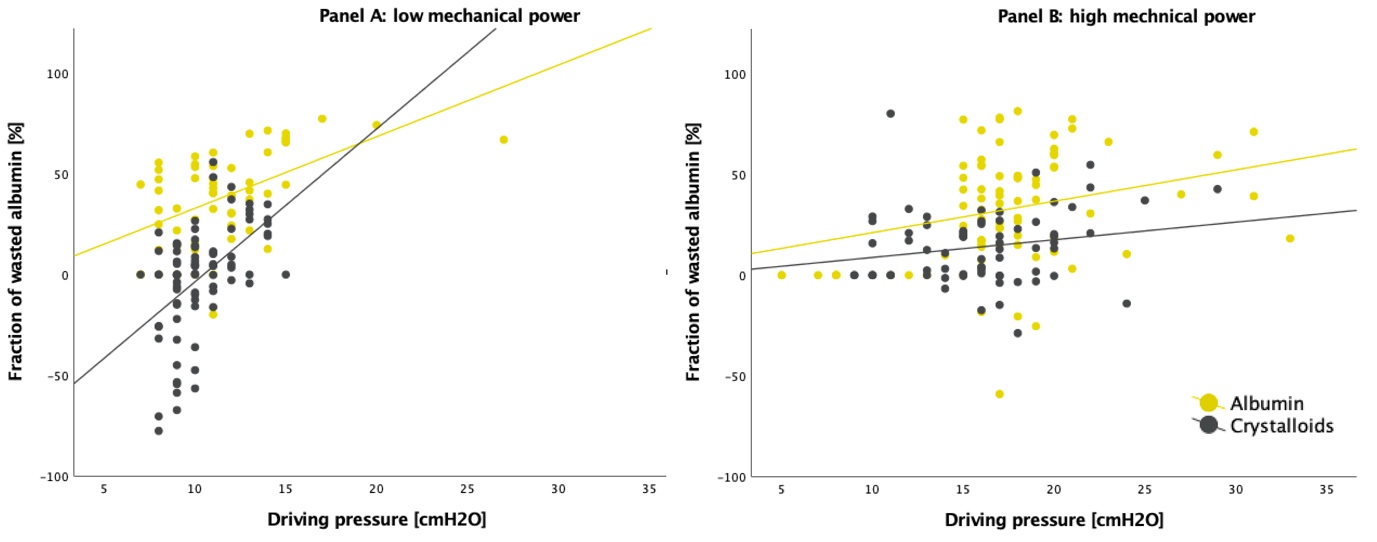


**References:**

S1) Percie du Sert N, Hurst V, Ahluwalia A, Alam S, Avey MT, Baker M, et al. The ARRIVE guidelines 2.0: Updated guidelines for reporting animal research. *PLoS Biol* 2020;18:e3000410

S2) Romitti F, Busana M, Palumbo MM, Bonifazi M, Giosa L, Vassalli F, et al. Mechanical power thresholds during mechanical ventilation: An experimental study. *Physiol Rep* 2022;10:e15225

S3) Serpa Neto A, Deliberato RO, Johnson AEW, Bos LD, Amorim P, Pereira SM, et al. Mechanical power of ventilation is associated with mortality in critically ill patients: an analysis of patients in two observational cohorts. *Intensive Care Med* 2018;44:1914-1922

S4) Gattarello S, Pasticci I, Busana M, Lazzari S, Palermo P, Palumbo MM, et al. Role of fluid and sodium retantion in experimental ventilator-induced lung injury. Fron Physiol 2021; 13:12:743153
